# The *Crit* coefficient in Mokken scale analysis: a simulation study and an application in quality-of-life research

**DOI:** 10.1007/s11136-021-02924-z

**Published:** 2021-09-02

**Authors:** Daniela R. Crișan, Jorge N. Tendeiro, Rob R. Meijer

**Affiliations:** 1 Department Psychometrics and Statistics, Faculty of Behavioral and Social Sciences, University of Groningen, Groningen, The Netherlands; 2 Education and Research Center for Artificial Intelligence and Data Innovation, Hiroshima University, Higashihiroshima, Japan

**Keywords:** Mokken scaling, MSA, *Crit*, Monotonicity, IIO, Item fit

## Abstract

**Purpose:**

In Mokken scaling, the *Crit* index was proposed and is sometimes used as evidence (or lack thereof) of violations of some common model assumptions. The main goal of our study was twofold: To make the formulation of the *Crit* index explicit and accessible, and to investigate its distribution under various measurement conditions.

**Methods:**

We conducted two simulation studies in the context of dichotomously scored item responses. We manipulated the type of assumption violation, the proportion of violating items, sample size, and quality. False positive rates and power to detect assumption violations were our main outcome variables. Furthermore, we used the *Crit* coefficient in a Mokken scale analysis to a set of responses to the General Health Questionnaire (GHQ-12), a self-administered questionnaire for assessing current mental health.

**Results:**

We found that the false positive rates of *Crit* were close to the nominal rate in most conditions, and that power to detect misfit depended on the sample size, type of violation, and number of assumption-violating items. Overall, in small samples *Crit* lacked the power to detect misfit, and in larger samples power differed considerably depending on the type of violation and proportion of misfitting items. Furthermore, we also found in our empirical example that even in large samples the *Crit* index may fail to detect assumption violations.

**Discussion:**

Even in large samples, the *Crit* coefficient showed limited usefulness for detecting moderate and severe violations of monotonicity. Our findings are relevant to researchers and practitioners who use Mokken scaling for scale and questionnaire construction and revision.

**Supplementary Information:**

The online version contains supplementary material available at 10.1007/s11136-021-02924-z.

## Introduction

Mokken scale analysis (MSA; e.g., [9, 12, 19, 20, 22]) is a popular item response theory (IRT) approach for evaluating the psychometric quality of tests and questionnaires in various fields such as psychology, education, health and quality-of-life (QoL), or marketing (e.g. [7, 11, 16, 33, 34]). Notable models within Mokken scaling include the monotone homogeneity model (MHM) and the double monotonicity model (DMM) [12]. Sijtsma and Molenaar [19] and Meijer and Tendeiro [9] offer a gentle introduction to Mokken scaling to those unfamiliar with MSA.

In empirical studies, Loevinger’s *H* coefficient [5, 6, 13] is the most popular method to evaluate the quality of a Mokken scale. However, there are other methods to check the assumptions of Mokken scaling (see e.g., [19, 30]). In this article, we focus on the so-called *Crit* coefficient [15] that summarizes information from the *H* coefficient and other statistics concerning the violation of model assumptions.

More than 20 years have passed since *Crit* was proposed [15]. Meanwhile, the coefficient has been mentioned in MSA tutorials and instructional modules as an overall critical value useful to assess violations of model assumptions (e.g., [21, 23, 30, 34]), and it is available in two software packages [15, 27, 28]. However, perhaps due to the general lack of insight around *Crit*, it is not routinely used in practical applications as an effect size for violations of the monotonicity (M) assumption of the MHM and for violations of the item invariant ordering (IIO) assumption of the DMM (e.g., [33]). There are no theoretical or empirical studies that provide a good insight into the definition of *Crit* and the basis for the suggested rules-of-thumb. Molenaar and Sijtsma [15] provided some tentative rules of thumb to help researchers interpret the severity of a violation, but these rules of thumb were *empirically* (i.e., not theoretically) derived from a limited set of real datasets.

To fill this gap, and to investigate whether *Crit* can be advocated to be used in practical applications, in the present study we first discuss the formulation of the *Crit* coefficient in the context of Mokken scale analysis and some of its properties. As we will discuss, the *Crit* coefficient is an empirically driven formula, thus justifying our interest in further understanding its *theoretical* basis. To that extent, we present the results of two simulation studies that investigate the distribution of the *Crit* coefficient under several measurement conditions. Furthermore, we present an empirical example concerning quality-of-life data, in order to link our simulations studies to empirical research and to show researchers how our findings may contribute to the interpretation of MSA applications in the field of QoL research. Finally, we discuss the usefulness of the *Crit* coefficient and of the proposed rules of thumb as a measure of effect size for violations of Mokken scales.

### The monotonicity assumption in MSA

In nonparametric IRT models, as in other item response theory models, it is assumed that the item response functions (IRFs) are monotonically nondecreasing (the monotonicity assumption, M for short). In this study we restrict ourselves to dichotomous items scored 0 (e.g., “incorrect” or “disagree”) or 1 (e.g., “correct” or “agree”). Then M means that the probability of a correct response (or the probability of endorsing the item) is a nondecreasing function of the latent trait or person characteristic that is measured (often denoted *θ*). In MSA, the so-called restscore (*R*_*−i*_), where *R*_*−i*_ is the number-correct score over all items *excluding* item *i*, is used as a proxy for a person’s value on the person characteristic of interest [15]. When M holds then it applies that, apart from sampling fluctuations [3],

1 $$P\left( {X_{i} = 1|R_{{ - i}} = s} \right) \ge P\left( {X_{i} = 1|R_{{ - i}} = r} \right), \quad {\text{for all}}\;s > r.$$

If this order does not hold in the sample, the item may violate the assumption of monotonicity.

**Fig. 1:**
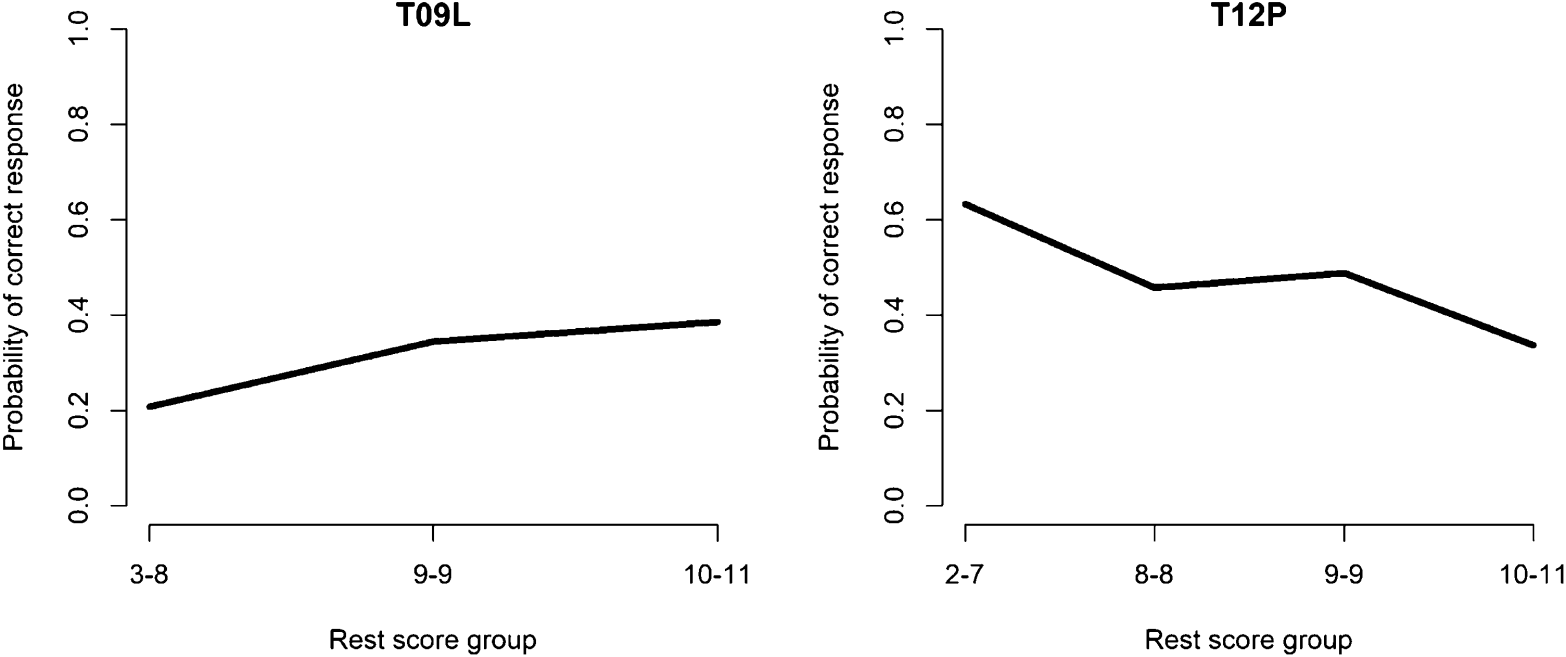
Estimated IRFs of two transitive reasoning items. Item on the left (T09L) is monotonically non-decreasing, and item on the right (T12P) shows violations of monotonicity

Figure 1 shows the estimated IRFs of two items from a transitive reasoning test [32] (data available in the “Mokken” package [27, 28]). Item T09L has a monotonically nondecreasing IRF, while item T12P indicates a violation of the monotonicity assumption.

We can check the M assumption for item *i* by comparing the probabilities of a correct response or of endorsement between all restscore groups *s* and *r* (*s* > *r*) and counting the number of times Eq. 1 does not hold. Then, the *Crit* coefficient for checking violations against monotonicity can be calculated as follows [15]:

2 $$\begin{aligned} Crit_{i} & = 50 \times \left( {0.30 - H_{i} } \right) + \sqrt {\# vi} + 100 \times \# vi/\# ac \\ & \quad + 100 \times maxvi + 10 \times \sqrt {sum} + 1000 \times sum/\# ac \\ & \quad + 5 \times zmax + 10 \times \sqrt {\# zsig} + 100 \times \# zsig/\# ac. \\ \end{aligned}$$

In Eq. 2, *H*_*i*_ is the scalability coefficient of item *i*, *#vi* denotes the number of violations (the number of times Eq. 1 does not hold), *#ac* denotes the total number of pairs of restscore groups that are being compared, *maxvi* is the size of the largest violation, *sum* denotes the sum of all violations, and, finally, *zmax* and *#zsig* refer to the normal deviates associated with each violation. An example of how to obtain all these quantities is available in the Online Resource.

Molenaar and Sijtsma [15] proposed the following rules of thumb for *Crit*:

1. A *Crit* coefficient larger than 80 casts serious doubt on the fit of the item to the model;
2. A *Crit* coefficient between 40 and 80 indicates that the evidence of a violation is unclear. However, some authors (e.g., [23]) interpret *Crit* ≥ 40 as evidence of a serious model violation;
3. Finally, a *Crit* coefficient lower than 40 indicates that there is no strong evidence in the data supporting the hypothesis of model misfit.

### The IIO assumption in MSA

The DMM implies invariant item ordering (IIO) [18]. This assumption implies that the ordering of the items according to the item difficulty or item proportion correct or item popularity is the same across all values of the person characteristic. In other words, the IIO assumption means that the IRFs do not intersect. If items are ordered and numbered from the most difficult (least popular) to the least difficult (most popular), then IIO implies that, apart from sampling fluctuations:

3 $$P\left( {X_{i} = 1{\text{|}}R_{{ - ij}} = r} \right) \le P\left( {X_{j} = 1{\text{|}}R_{{ - ij}} = r} \right), \quad {\text{for all}}\;r,$$

where *R*_*−ij*_ is the number-correct score over all items *excluding* items *i* and *j*. If Eq. 3 does not hold in the sample, then at least some items may violate the assumption of invariant item ordering.

**Fig. 2:**
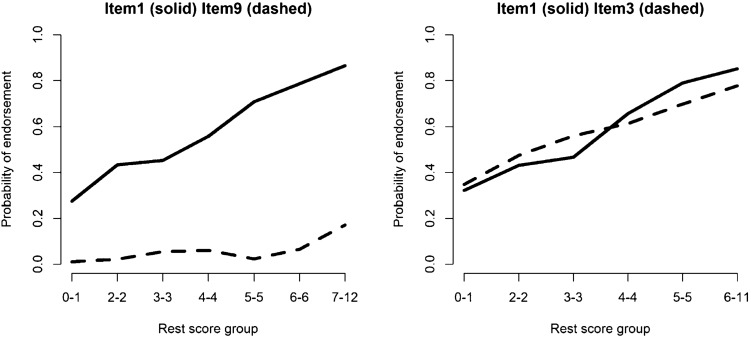
Estimated item response functions of two pairs of items. Items 1 and 9 (on the left) do not intersect, and items 1 and 3 (on the right) intersect

To illustrate this, Fig. 2 shows the estimated IRFs of two pairs of dichotomized items from a questionnaire asking participants about their strategies of coping with industrial malodour [1] (data available within the “Mokken” package [27, 28]). Items 1 (“keep windows closed”) and 9 (“file complaint with producer”) do not intersect, while items 1 and 3 (“search source of malodor”) do. IIO is being violated here because the relative popularity of items 1 and 3 switches across the restscore groups.

Similarly as for checking monotonicity, for IIO the *Crit* coefficient can be calculated for each item, according to Eq. 2, and the same rules of thumb apply [15]. An example of how to obtain the quantities in Eq. 2 when evaluating IIO is available in the Online Resource.

### Aim of the study

Clearly, Eq. 2 is a complex weighted sum of various features of the data. Importantly, the weights and the advised rules of thumb are very unclear. More than 20 years have passed since *Crit* was proposed and little attention has been given to understanding its functioning. Below we show the results of two simulation studies to further understand the *Crit* coefficient. Study 1 addresses the following research questions: (RQ1A) How is the *Crit* coefficient for assessing M distributed under model-fitting data and to what extent is *Crit* sensitive to scale quality and sample size? (RQ1B) How is the *Crit* coefficient for assessing M distributed under different types of M violations and to what extent is the distribution of *Crit* affected by the number of model-violating items and sample size? Study 2 addresses similar research questions as Study 1 but with a focus on IIO, that is: (RQ2A) How is the *Crit* coefficient for assessing IIO distributed under model-fitting data and to what extent is *Crit* sensitive to scale quality and sample size? (RQ2B) How is *Crit* distributed when violations of IIO occur and to what extent is it affected by the number of model-violating items and sample size? Specifically, we were interested in the false positive and true positive (power) rates of the *Crit* coefficient when following the rules of thumb proposed by Molenaar and Sijtsma [15]. We also investigated how *Crit* compares to another, more conventional method of investigating violations of M and of IIO: checking whether there is one or more significant violations of M or of IIO. Furthermore, we re-analyzed a dataset from an empirical quality-of-life study and interpreted the results in light of the findings from our simulation studies.

## Simulation setup

The first study was aimed at determining the distribution of the *Crit* coefficient under (violations of) monotonicity, and the second study was aimed at determining the distribution of the *Crit* coefficient under (violations of) IIO. More specifically, in each study we generated both model-fitting and model-misfitting data. We then computed the *Crit* coefficient according to Eq. 2. For RQ1A we used a 3 (Scale quality) × 3 (Sample size) fully crossed design, resulting in 3 × 3 = 9 conditions. For RQ1B we simulated data according to a 3 (Type of *M* violation) × 3 (Number of misfitting items) × 3 (Sample size) fully crossed design, resulting in 3 × 3 × 3 = 27 conditions. Together, the 36 conditions constitute the design of the first simulation study (Study 1).

Similarly, for Study 2, RQ2A we used a 3 (Scale quality) × 3 (Sample size) fully crossed design, resulting in 3 × 3 = 9 conditions. For RQ2B we simulated data according to a 2 (Type of IIO violation) × 3 (Number of misfitting items) × 3 (Sample size) fully crossed design, resulting in 2 × 3 × 3 = 18 conditions. Together, the 27 conditions form the design of the second simulation study (Study 2).

Additionally, the number of significant violations (*#zsig*) of the M and the IIO assumptions was computed and the results were compared to those based on *Crit* [15]. In all conditions, we generated dichotomous item responses (coded 0/1) on *I* = 10 items. Many Mokken studies analyze relatively short scales; These scales are sometimes part of a larger test, survey, or inventory that has a more complex structure (e.g., see [8, 10] for such analyses using clinical and personality scales between 5 and 13 items). For each condition we generated 1000 replications. Below we provide details about the independent and the outcome variables. For readers who are interested in replicating our results, we included detailed information on the data generating processes in the Online Resource.

### Independent variables

We varied four factors in each of the two simulation studies: type of violation, number of assumption-violating items (*I*_*misfit*_), scale quality, and sample size (*N*).

#### Type of violation

**Fig. 3:**
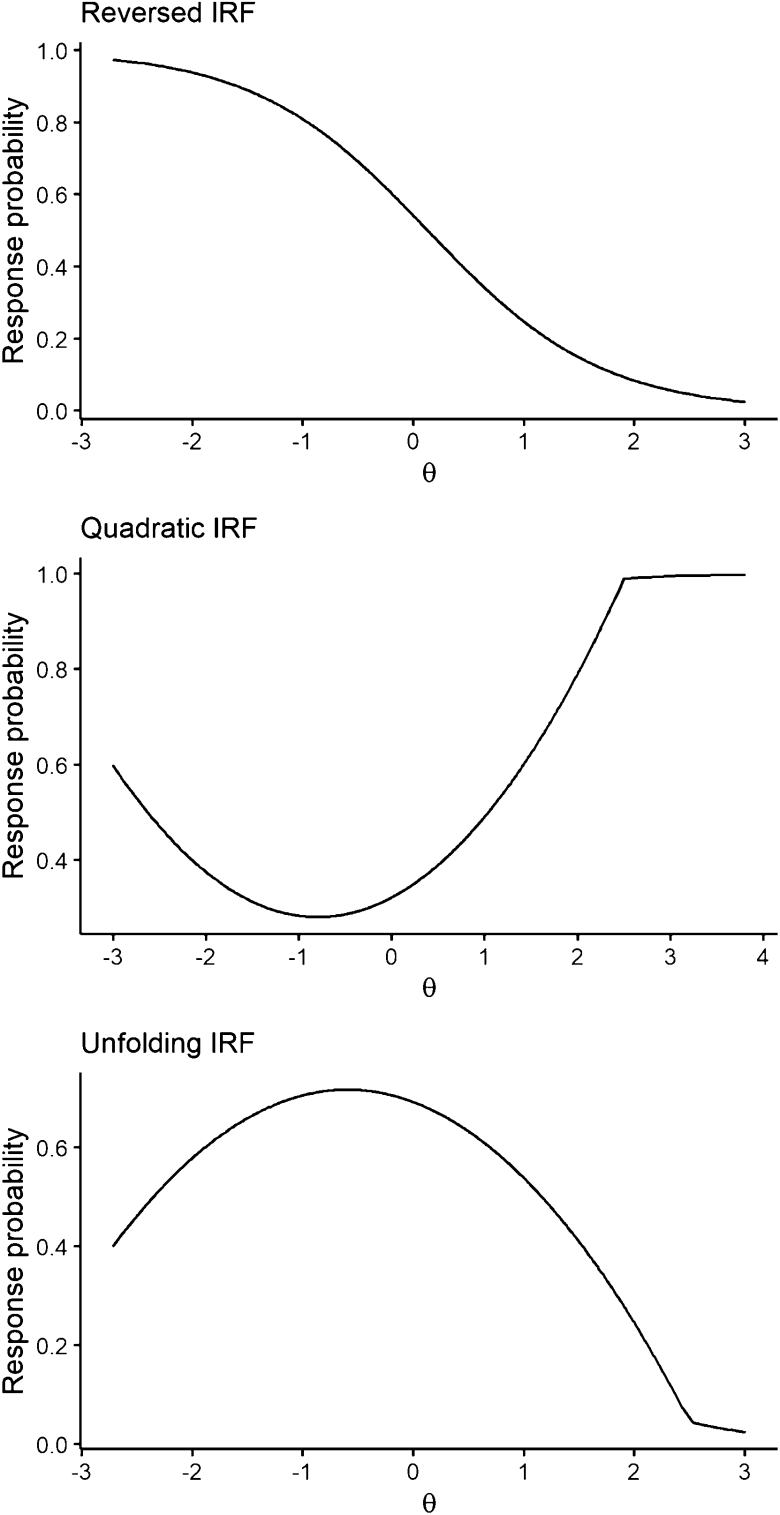
Examples of violations of monotonicity through reversed, quadratic, and unimodal IRFs

**Fig. 4:**
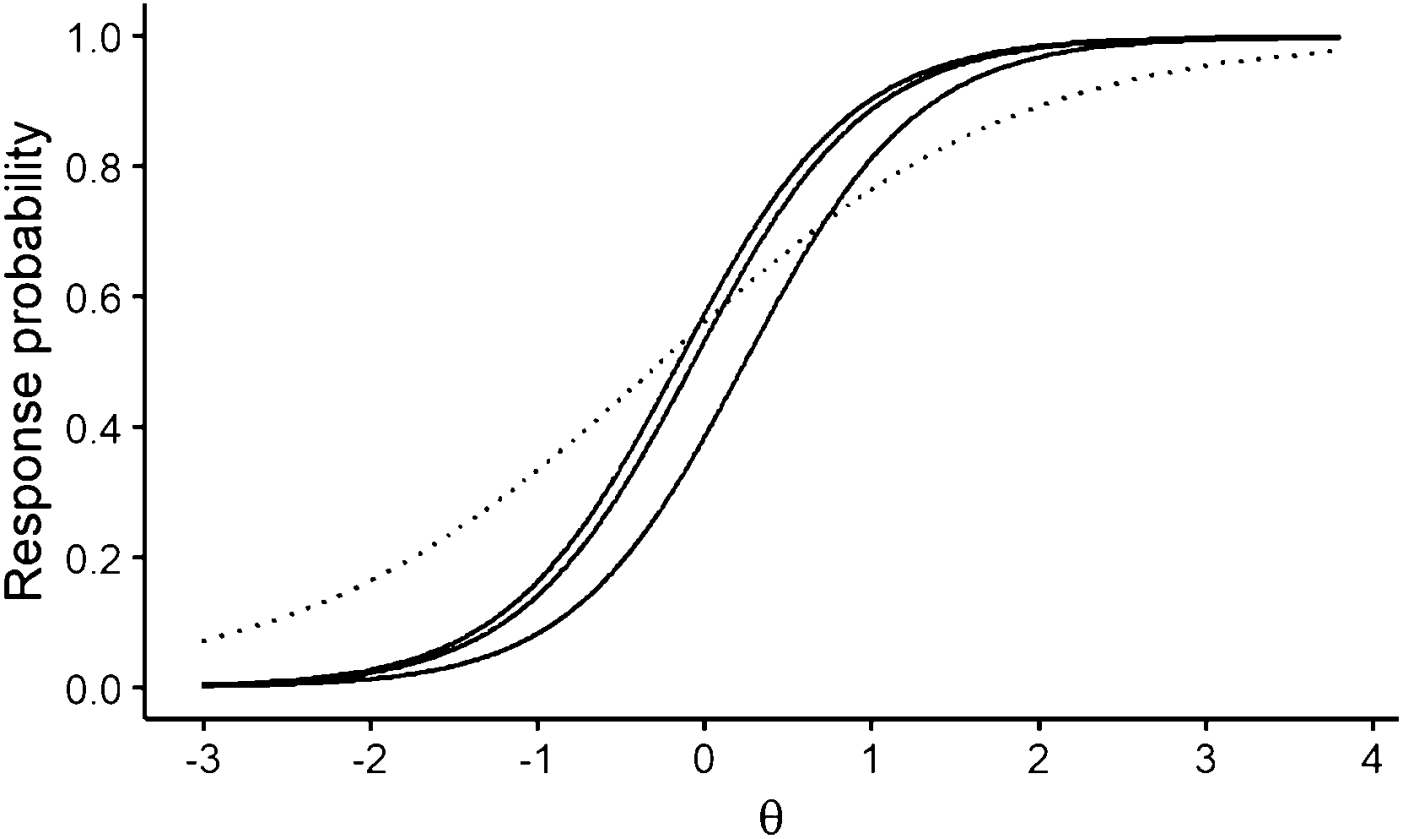
Example of violations of IIO through intersecting IRFs. In this plot, the dotted IRF violates the IIO assumption by intersecting with the solid IRFs

This factor was operationalized differently across studies. In Study 1, violations of M were introduced by generating reversed, unimodal, or quadratic IRFs, as described in the Online Resource and illustrated in Fig. 3. Reversed IRFs are seldom encountered in practice, as these items are either reverse-coded or removed in the early stages of scale construction. Nevertheless, it is interesting to see how model-fitting items are affected by the presence of items that have been, say, coded improperly. In Study 2, the *I*_*misfit*_ items were generated to intersect with the remaining *I* − *I*_*misfit*_ items by setting their slope either higher or lower than the common slope of all fitting items (see the Online Resource for details and Fig. 4 for an illustration).

#### Number of assumption-violating items

We considered three values for *I*_*misfit*_ for both studies: 1, 3, and 5. Thus, either 10%, 30%, or 50% of the items in the scale were violating either the M or the IIO assumption.

#### Scale quality

We only manipulated this factor in the model-fitting conditions of Study 1 and Study 2. In Study 1 we did this because when the IRFs have a different shape than monotone nondecreasing, it is not clear if the guidelines for scale quality proposed by Mokken [12] still hold. In Study 2 we did this to be consistent. Through a process of trial-and-error, we obtained Mokken scales of varying quality as reflected by the *H* coefficient for the entire scale [12]: Medium and strong scales (*H* ≥ 0.4), weak scales (0.3 ≤ *H* < 0.4), and scales where the *H* coefficient was smaller than 0.3, that is, where the items did not form a Mokken scale (unscalable). See Table C1 in the Online Resource for the parameters we used to obtain these Mokken scales.

**Table 1:** False positive rates and power for the *Crit* coefficient for violations of M

Type of violation	False positive rates^a^			True positive rates (power)^b^		
	*I*_*misfit*_ = 1	*I*_*misfit*_ = 3	*I*_*misfit*_ = 5	*I*_*misfit*_ = 1	*I*_*misfit*_ = 3	*I*_*misfit*_ = 5
Quadratic IRFs						
*N* = 100	< 0.1	< 0.1	< 0.1	1.2	0.7	0.5
*N* = 500	< 0.1	< 0.1	0.1	12.1	9.8	6.5
*N* = 1000	< 0.1	< 0.1	< 0.1	10.7	6.7	4.9
Unimodal IRFs						
*N* = 100	< 0.1	< 0.1	0.3	5.2	4.0	1.8
*N* = 500	< 0.1	0.2	5.6	99.2	97.6	78.7
*N* = 1000	< 0.1	0.1	5.4	99.5	99.6	91.3
Reversed IRFs						
*N* = 100	< 0.1	< 0.1	2.0	7.2	4.9	1.9
*N* = 500	< 0.1	1.7	81.5	99.8	99.9	80.6
*N* = 1,000	< 0.1	3.1	87.5	100.0	100.0	86.7

Values shown are percentages of Crit values at least equal to 80

^a^Values computed over the (*I* − *I*_*misfit*_) items

^b^Values computed over the *I*_*misfit*_ items

#### Sample size

The statistical significance of violations is also part of the computation of *Crit*. The last three terms in Eq. 2 contain the normal deviates associated with each violation (*zmax*) and their probability of exceedance or statistical significance (*#zsig*). With very large sample sizes, even small deviations from the null model are statistically significant and thus contribute to the *Crit* coefficient. Therefore, in both studies, we determined the distribution of *Crit* under three sample sizes: *N* = 100, 500, and 1000, representing small, medium, and large samples found in many empirical Mokken studies [14, 31].

### Outcome variable

In each simulation study we computed the *Crit* coefficient for all items according to Eq. 2. We then plotted the distribution of the *Crit* coefficient separately for each type of misfit, *N*, and *I*_*misfit*_, and we computed the false positive and true positive (power) rates. The false positive rate was defined as the percentage of cases in which an item was generated to comply with the model but was detected as misfitting (i.e., had a *Crit* ≥ 80). The true positive rate, or the power of *Crit* to detect misfit, was defined as the percentage of cases in which an item was correctly detected as misfitting, that is, the item was generated to violate the M or the IIO assumption and had a *Crit* ≥ 80.

We also calculated the number of significant violations of M or IIO (*#zsig*) for all items according to [15]. We then computed false positive and power rates for *#zsig* separately for each type of misfit, *N*, and *I*_*misfit*_. The false positive rate was defined as the percentage of cases in which *#zsig* > 0 even though the item was generated to comply with the model. Power was calculated as the percentage of cases in which *#zsig* > 0 and the item was generated to violate M or IIO. For the analyses we set *minvi* equal to 0.03 and *minsize* equal to *N*/10 for $N \ge 500$ and to ${\text{max}}\left( {N/3,50} \right)$ for *N* = 100.

### Implementation

We implemented the simulation in R [17] and used the “Mokken” package [27, 28] to compute the *Crit* coefficient and the *#zsig* values for monotonicity and invariant item ordering. All R script files and generated output files are open and available at https://osf.io/eh2my/.

## Simulation results

### *Crit* for violations of monotonicity

We first present the results concerning false positive rates and power for the *Crit* coefficient when evaluating the assumption of monotonicity, and how they relate to those of *#zsig*. Concerning RQ1A, we found that the overall false positive rates (i.e., calculated over all 10 items in the 9 conditions with *I*_*misfit*_ = 0) were very low, with only 0.01% of the *Crit* values above 80. Moreover, the distribution of *Crit*, which had a median value of 0 and an interquartile range (IQR)^1^ of 0, was not affected by either scale quality or sample size. *Crit* values above 0 are most likely random fluctuations. Regarding the false positive rates of *#zsig* in the *I*_*misfit*_ = 0 conditions, we found that 0.09% of the values were larger than 0.

To answer RQ1B, Table 1 shows the power and the false positive rates of the *Crit* coefficient, and Fig. E1 in Online Resource depicts the distribution of *Crit* under the different types of M violation, separately for *I*_*misfit*_ and *N*.

We found that *Crit* for checking the monotonicity assumption in MSA was affected to a large extent by sample size, the number of misfitting items, and the type of violation of monotonicity: For small samples (*N* = 100), *Crit* had very low power to detect violations of monotonicity, regardless of the type or amount of violation. In larger samples (*N* = 500, 1,000), the power of *Crit* improved depending on the number of misfitting items and type of M violation, with the highest values for unimodal and reversed IRFs (between 80 and 100%). For most conditions studied, false positive rates were relatively low, but they increased with number of misfitting items and sample size. This is because the *H* value, which is a rescaled inter-item covariance [14, 19], is part of the computation of *Crit*, and because in large samples, even trivial violations can become statistically significant, contributing to the computation of *Crit* (Eq. 2).

We found a very similar pattern of results concerning the false positive and power rates of *#zsig* for violations of M (see Table D1 in Online Resource). A visual comparison of Table 1 and Table D1 reveals that the two methods, *Crit* and *#zsig*, performed similarly with respect to the false positive rates and power to detect violations of M in most simulation conditions. In large samples and with quadratic IRFs, the power of *#zsig* was slightly higher than for *Crit*, but remained very low nonetheless. When *I*_*misfit*_ = 5, *#zsig* had a substantially lower power than *Crit* for unimodal IRFs (*N* = 500, 1,000) and for reversed IRFs (*N* = 500).

### *Crit* for violations of IIO

For violations of IIO, we also computed the false positive rates and power of *Crit* and *#zsig*, defined similarly as above. In Table 2 as well as in Fig. E2 of the Online Resource, we depicted the results pertaining to our research questions RQ2A and RQ2B. The findings were similar as for Study 1. The nature of IIO violations, however, made it difficult to distinguish between fitting and misfitting items: When the *I*_*misfit*_ IRFs intersected with the IRFs of the (*I* − *I*_*misfit*_) items, the latter were considered misfitting as well. This is because the *Crit* coefficient for item *i* is a summary of, among other quantities, how many times Eq. 3 does not hold in the sample for each pair formed by item *i* with the remaining items. This led to high false positive rates for the fitting items in the misfit conditions. Consequently, it made little sense to interpret false positive rates for the fitting items in the misfit conditions. Therefore, we only interpreted the false positive rates in the conditions with *I*_*misfit*_ = 0 (RQ2A) and the power of *Crit* to detect misfit in the conditions in which *I*_*misfit*_ = 1, 3, 5 (RQ2B). We compared the false positive rates and power of *Crit* with the values we obtained for *#zsig* (Table D2 in the Online Resource).

**Table 2:** False positive rates (top panel) and power (lower panel) for the *Crit* coefficient for violations of IIO

	*N* = 100	*N* = 500	*N* = 1,000
^a^False positive rates			
Scale quality			
Unscalable items	3.2	0.4	0.1
Weak scales	2.0	< 0.1	< 0.1
Medium-strong scales	1.7	< 0.1	< 0.1
^b^Power			
Number of violating items			
*I*_*misfit*_ = 1	6.0	20.9	29.3
*I*_*misfit*_ = 3	5.4	16.2	22.3
*I*_*misfit*_ = 5	4.6	10.3	15.0

Values shown are percentages of *Crit* values at least equal to 80

^a^Values computed over the *I* items in the *I*_*misfit*_ = 0 conditions (9 conditions)

^b^Values computed over the *I*_*misfit*_ items in the *I*_*misfit*_ = 1, 3, 5 conditions (18 conditions)

We found that the *Crit* coefficient for assessing violations of IIO has lower false positive rates (RQ2A) and higher power (RQ2B) in larger samples compared to small samples. Regarding the power of *Crit*, we found the same effects of *N* and *I*_*misfit*_ as for violations of monotonicity, though the overall power for detecting violations of IIO was considerably lower (up to only 30%). Higher power was obtained in larger samples because violations became statistically significant, whereas a decrease in power with relatively many misfitting items was due to lower inter-item correlations (and thus lower *H*_*i*_ values).

Regarding *#zsig* for violations of IIO (Table D2 in the Online Resource), we again found similar results as for *Crit* in terms of false positive rates and power. The power of *#zsig* to detect violations of IIO increased with *N* but, as opposed to *Crit*, it also increased with *I*_*misfit*_. Consequently, for many misfitting items (*I*_*misfit*_ = 5) and large samples (*N* = 500, 1,000), *#zsig* had considerably higher power to detect misfit compared to *Crit*. Nonetheless, the power of *#zsig* are still low (29.8% for *N* = 500 and 52.0% for *N* = 1,000).

## Empirical example: mental health

To illustrate the findings above we analyzed data from the General Health Questionnaire (GHQ-12; [2]). GHQ-12 is a self-administered questionnaire consisting of 12 items, each measuring the severity of a mental problem over the past several weeks on a 4-point Likert scale. High scores indicate worse mental health. The data we used came from Wave 10 of the Understanding Society study, also known as the United Kingdom Household Longitudinal Study (UKHLS; [26]), the largest longitudinal household panel study in the UK. The dataset we analyzed consisted of the responses of 18,444 adult respondents to the GHQ-12.^2^ Records containing missing data on any of the GHQ-12 items were removed. The first column of Table 3 shows a short version of the GHQ-12 item content. We dichotomized the item responses: the scores “1” and “2” were recoded as “0” and the scores “3” and “4” were recoded as “1”. Also, to avoid issues due to nested data, we randomly sampled a single member from each household in our final dataset. Dichotomizing the item responses and selecting one member per household is an appropriate solution in this methodological context, where the aim was to illustrate some properties of the *Crit* coefficient on non-clustered, binary data. From a substantive perspective this approach might not be ideal, as it causes loss of information. For researchers who wish to analyze such data using Mokken scale analysis, we refer to Koopman et al. [4], who proposed point estimates, standard errors, and test statistics for scalability coefficients for nested data. These authors incorporated their proposed methods into what they called a two-step, test-guided MSA procedure for scale construction.

**Table 3:** Results from the invariant item ordering checks for the GHQ-12 items

Item	ItemH	*#ac*	*#vi*	*#vi/#ac*	*maxvi*	*sum*	*sum/#ac*	*zmax*	*#zsig*	*Crit*
1. Able to concentrate	0.51	33	2	0.06	0.08	0.11	0.0034	6.86	2	65
2. Loss of sleep over worry	0.48	33	2	0.06	0.10	0.18	0.0053	8.74	2	81
3. Playing a useful role	0.51	33	2	0.06	0.08	0.12	0.0035	7.47	2	69
4. Capable of making decision	0.58	33	0	0.00	0.00	0.00	0.0000	0.00	0	0
5. Felt constantly under strain	0.60	33	1	0.03	0.05	0.05	0.0014	5.03	1	35
6. Couldn’t overcome difficulties	0.59	33	2	0.06	0.08	0.16	0.0048	7.47	2	67
7. Able to enjoy day-to-day activities	0.56	33	0	0.00	0.00	0.00	0.0000	0.00	0	0
8. Able to face problems	0.62	33	0	0.00	0.00	0.00	0.0000	0.00	0	0
9. Feeling unhappy and depressed	0.64	33	1	0.03	0.05	0.05	0.0014	5.03	1	33
10. Losing confidence	0.58	33	1	0.03	0.08	0.08	0.0023	6.86	1	49
11. Thinking of self as worthless	0.63	33	0	0.00	0.00	0.00	0.0000	0.00	0	0
12. Feeling reasonably happy	0.59	33	3	0.09	0.10	0.17	0.0052	8.74	3	85

In order to ensure that higher (item) scores reflect more severe mental health issues, the item response functions should be monotonically non-decreasing across the entire range of mental health levels. One research question was to assess whether this assumption of monotonicity was supported by the data and, if not, to determine the severity of these violations. We also investigated whether the GHQ-12 items had the same order with respect to endorsement proportions across different values of the mental health symptom severity. Thus, the research question here was whether this assumption of invariant item ordering was supported by the empirical data.

To investigate these questions, we conducted MSA on these data and we inspected the *Crit* coefficient for M and IIO. For *minvi* the default setting was used, that is *minvi* = 0.03 [27]. For *minsize*, the default setting when $N \ge 500$ is *N*/10, thus *minsize* = 1844. Given these settings, the number of comparison groups was equal to 4 for both M and IIO.

**Fig. 5:**
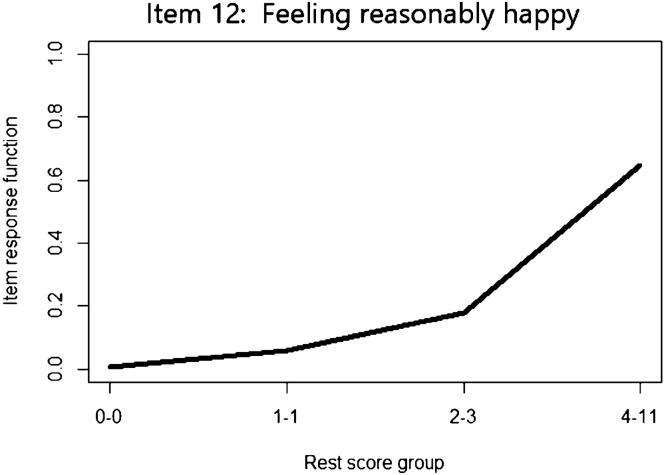
Estimated IRF, indicating no evidence of violations of the M assumption

There was no evidence that any of the items violated the M assumption – the *Crit* coefficient was equal to 0 for all items as there were no violations that were statistically significant or larger than *minvi*. The estimated IRFs did not indicate any violations either (see Fig. 5 for an example).

Thus, to answer the research question pertaining to monotonicity, the following can be concluded: Given the large sample size (*N* = 18,444), there is no strong evidence of GHQ-12 items that exhibit violation of the monotonicity assumption. In light of our simulation results, we are fairly confident that patterns such as unimodal or reversed IRFs are very unlikely. However, given the low power of *Crit* to detect quadratic IRFs, we could not draw a strong conclusion regarding this type of M violation.

**Fig. 6:**
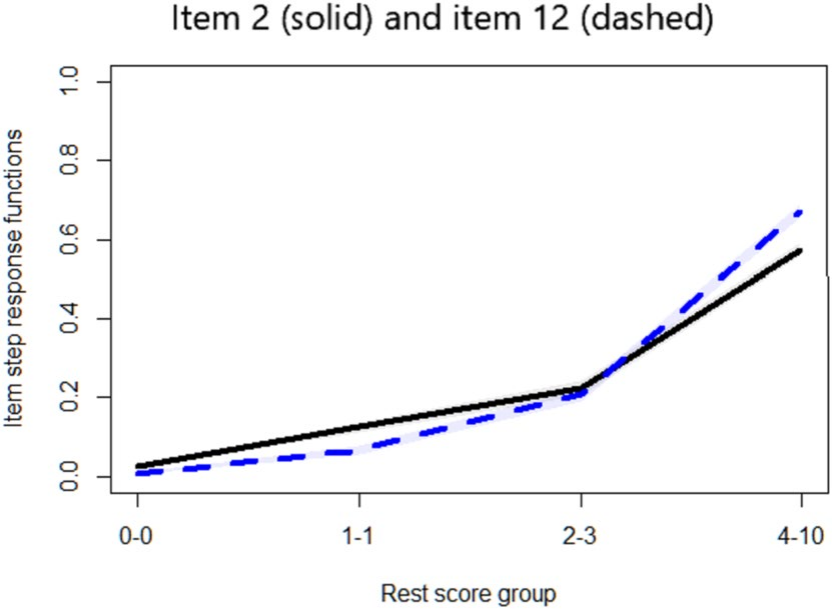
Item-pair estimated IRFs illustrating violations of IIO

With respect to violations of IIO, Table 3 shows that there were two items (items 2 and 12) with several statistically significant violations and with *Crit* coefficient larger than 80, indicating the presence of violations of IIO. A plot illustrating these violations is shown in Fig. 6.

Based on our simulation results for the combination of large sample size and high scale *H* coefficient, we expect the false positive rate for *Crit* to be very low (< 0.1%) and the power to be between 22.3 and 29.3%. Thus, there was strong evidence that the two items with large *Crit* values indeed exhibited violations of IIO. We further observed that after dropping items 12 and 2 one by one from the scale, the *Crit* values for the remaining items decreased below the threshold 80. Thus, a researcher may combine this information from the *Crit* coefficient together with information from the estimated IRFs to conclude that items 2 and 12 violated IIO.

## Discussion

In this study we discussed and investigated characteristics of the *Crit* coefficient, an ambiguously defined index of violations of common assumptions in Mokken scale analysis, which is sometimes used in applications of MSA in QoL research. We presented both the formulation of *Crit* and discussed several characteristics of the coefficient (see also Online Resource). We conducted two simulation studies using dichotomously scored item responses, in which we investigated the distribution of *Crit* under various measurement conditions, its power, and false positive rates. For a thorough understanding of the usefulness of the *Crit* coefficient, we compared its false positive and power rates with those of a more conventional method for assessing assumptions violations: whether or not there is one or more statistically significant violations of M or IIO (*#zsig*). Finally, we discussed an application of the *Crit* coefficient on QoL empirical data.

With respect to the distribution of the *Crit* coefficient when estimated using model-fitting data we found nominal false positive rates (i.e., less than 5%) in all conditions, both for monotonicity and for nonintersection, which were not affected by scale quality or sample size. When violations of monotonicity were introduced, we found considerably larger false positive rates for reversed IRFs when *Crit* was estimated in large samples with many model-violating items. With respect to the power of *Crit* we found that in small samples the power was very low, for both monotonicity and nonintersection. For unimodal and reversed IRFs and in larger samples power increased considerably, but it remained very low for detecting other types of violations of MSA assumptions.

Regarding the performance of *Crit* compared to more conventional methods, such as *#zsig*, our studies show that the latter did not consistently outperform the former in terms of false positive rates and power to detect misfit. Only in large samples and with many misfitting items, *#zsig* showed considerably higher power to detect violations of invariant item ordering.

A more detailed analysis of the results showed that the low power of *Crit* in small samples can be explained by the small number of restscore groups that met the minimum requirement in terms of size. Violations were masked by having too few restscore groups to take into account when calculating the response probabilities (Eqs. 1 and 3). In contrast, in larger samples (e.g., of size 500 or 1000) this became less of an issue. Finally, the low power of *Crit* to detect violations of nonintersection when half of the items were violating this assumption can be explained by the observation that, when relatively many items in a scale intersect with each other, the overall order of the items according to their probability of a correct response/endorsement becomes unclear and unstable.

Our simulation studies could be extended by considering different item formats (e.g., polytomous items or mixed-format items) and other ways of simulating violations of assumptions. For example, violations of monotonicity could be introduced by fitting a polynomial extension of the two-parameter logistic model (see [24, 25]).

### Take-home message

In light of the findings and of the conclusions outlined above, we have a number of practical suggestions when using the *Crit* coefficient. Practitioners of scale construction or scale revision should be cautious when using the *Crit* coefficient, as it has limited usefulness for detecting violations of monotonicity or invariant item ordering in practice. In general, Mokken scaling using small samples is not recommended [31]. This was also reflected in our simulation study. Violations of assumptions were masked by having too few restscore groups when calculating *Crit*. One solution may be to change the default settings used by most software packages (e.g., *minsize*), but then results become unstable due to too few observations per restscore group. Molenaar and Sijtsma [15] recommend conducting a sensitivity analysis by running the MSA with different values for *minsize* and checking whether the results differ substantially. If they do, then one should not draw strong conclusions, due to the instability of the results. However, even in large samples, the *Crit* coefficient may fail to detect violations of monotonicity.

Regarding the *Crit* coefficient for violations of IIO, the index had low power and it did not discriminate well between fitting and misfitting items (at least when IIO is evaluated using the ‘restscore’ method). Perhaps this feature can be more rightfully ascribed to the very nature of the IIO problem instead of to *Crit*. Indeed, an intersection between two IRFs implies a mutual interplay between pairs of items, and pinpointing one of the two items as ‘misfitting’ is more difficult than identifying violations of monotonicity. One solution may be to start with the item(s) that cause(s) the most violations with other items, that is, the items with the highest *Crit* coefficient [15]. This approach was illustrated in our empirical example: Dropping the two items with the largest *Crit*, one by one, led to an improvement of the outcomes.

To conclude, we suggest that the estimation of *Crit* should always be accompanied by a visual inspection of the estimated IRFs (e.g., [29]) and, if necessary, assumption-violating items should be removed one at a time, starting with the one that has the largest *Crit* [15], as we showed in our empirical example. We defend using a combination of approaches to data analysis as it is safer than overreliance on one single statistic, be it *Crit* or any other. Also, it is important to bear in mind that *Crit* performs best with large sample sizes. Nevertheless, this study offers a deeper understanding of the *Crit* coefficient and how it can be used in practice. It is our hope that practitioners feel now better equipped to utilize this particular tool in Mokken scale analysis.

## Supplementary Information

Below is the link to the electronic supplementary material.

Supplementary file1 (DOCX 283 kb)

## Data Availability

All the code used for the simulation studies is freely available at https://osf.io/eh2my/.

## Footnotes

1 Computed as the difference between the 75th and the 25th quantiles, it is a measure of statistical dispersion.

2 The data can be obtained upon request from the UK Data Service (https://ukdataservice.ac.uk/).
