# Supplementary material for: The Crit coefficient in Mokken scale analysis: a simulation study and an application in quality-of-life research
Source: Qual Life Res. 2021 Sep 2;31(1):49–59. doi: 10.1007/s11136-021-02924-z (PMC8800923; doi:10.1007/s11136-021-02924-z)
Supplement: Supplementary file 1 — Supplementary file1 (DOCX 283 kb) [file 11136_2021_2924_MOESM1_ESM.docx]

**Supplemental Material**

The *Crit* Coefficient in Mokken Scale Analysis: A Simulation Study and an Application in Quality-of-Life Research

Daniela R. Crișan^1^

Jorge N. Tendeiro^1,2^

Rob R. Meijer^1^

^1^University of Groningen

^2^Hiroshima University

1. **Monotonicity Checks and the *Crit* Value. An Example**

To illustrate how the monotonicity assumption is checked we use an example based on an analysis with the “mokken” package [7, 8]. The data set consisted of the dichotomous scores (0 = ‘incorrect’, 1 = ‘correct’) of 425 children on 12 items that measure transitive reasoning [9]. The data set “transreas” is freely available from the package “mokken”. The analyses were conducted with the default settings, that is, *minvi* was set to 0.03 and *minsize* was set to 85 [4]. Table A1 shows the summary table of the monotonicity checks for all items. Note that when *#vi* = 0, *Crit* is automatically set to 0 both in the MSP5 [4] and in the “mokken” [7, 8] software packages^[[1]](#footnote-1)^ for Mokken scale analysis. Also, the *Crit* value resulting from application of the formula is rounded down to the nearest integer. To understand the *Crit* value it is important to first discuss the elements in this table. This discussion is based on the explanations provided by Molenaar and Sijtsma [4].

**The elements of the *Crit* value.** The first column in Table A1 provides the names of the 12 items. The second column provides the numerical values of the item scalability coefficients (*H_i_*). Columns 3 through 9 provide information about violations of monotonicity.

Table A1

*Summary Output for Monotonicity Checks*

| **Item** | **ItemH** | **#ac** | **#vi** | **#vi/#ac** | **maxvi** | **sum** | **sum/#ac** | **zmax** | **#zsig** | **Crit** |
| --- | --- | --- | --- | --- | --- | --- | --- | --- | --- | --- |
| T09L | 0.17 | 3 | 0 | 0.00 | 0.00 | 0.00 | 0.0000 | 0.00 | 0 | 0 |
| T12P | -0.14 | 6 | 5 | 0.83 | 0.30 | 0.89 | 0.1481 | 4.13 | 4 | 401 |
| T10W | 0.14 | 6 | 0 | 0.00 | 0.00 | 0.00 | 0.0000 | 0.00 | 0 | 0 |
| T11P | -0.03 | 6 | 3 | 0.50 | 0.07 | 0.20 | 0.0328 | 0.88 | 0 | 116 |
| T04W | 0.05 | 3 | 0 | 0.00 | 0.00 | 0.00 | 0.0000 | 0.00 | 0 | 0 |
| T05W | 0.09 | 3 | 0 | 0.00 | 0.00 | 0.00 | 0.0000 | 0.00 | 0 | 0 |
| T02L | 0.08 | 3 | 0 | 0.00 | 0.00 | 0.00 | 0.0000 | 0.00 | 0 | 0 |
| T07L | 0.18 | 3 | 0 | 0.00 | 0.00 | 0.00 | 0.0000 | 0.00 | 0 | 0 |
| T03W | 0.19 | 3 | 0 | 0.00 | 0.00 | 0.00 | 0.0000 | 0.00 | 0 | 0 |
| T01L | 0.21 | 3 | 0 | 0.00 | 0.00 | 0.00 | 0.0000 | 0.00 | 0 | 0 |
| T08W | 0.28 | 2 | 0 | 0.00 | 0.00 | 0.00 | 0.0000 | 0.00 | 0 | 0 |
| T06A | 0.40 | 1 | 0 | 0.00 | 0.00 | 0.00 | 0.0000 | 0.00 | 0 | 0 |

To further explain these columns, consider the second item (T12P). Table A2 presents the detailed results of the monotonicity check for item T12P. There are 425 respondents grouped according to their restscore (*R_-i_* = *R_-T12P_*_,_ *i* = T12P in this case) on all the items excluding item T12P. In this case the restscore can take values from 0 to 11, so there are 12 restscore groups. To avoid counting violations that are either very small or that occur purely by chance, adjacent restscore groups are joined such that the total number of valid observations in each group is larger than a preset criterion called *minsize*, and only violations greater than a preset criterion called *minvi* are counted [4]. In our case, the minimum number of participants in the group (denoted *minsize)* was set here at the default value of 85 and because this *minsize* was not met by all 12 restscore groups, some groups were combined, resulting in 4 restscore groups (column ‘Group’ in the output depicted in Table A2). The first group contained respondents with restscores 2 to 7, the second group contained respondents with a restscore of 8, the third group contained respondents with a restscore of 9, and the fourth group contained respondents with restscores 10 to 11 (columns ‘Lo Score’ and ‘Hi Score’ in the output in Table A2). Thus, restscores 2 through 7 and 10 through 11 had to be combined in order to achieve *minsize* = 85. The reason restscores 0 and 1 are not in the table is that there were no respondents with these scores.

Column ‘N’ in Table A2 shows that there are 98 respondents with a restscore between 2 and 7, 94 respondents with a restscore 8, and so on. Next, for each restscore group we counted how many respondents scored 0 (incorrect answer) on this item and how many scored 1 (columns marked ‘F 0’ and ‘F 1’ in Table A2). Under the assumption of monotonicity we expect these proportions to be nondecreasing between adjacent restscore groups. A decrease in proportion-correct between restscore groups may be indicative for a violation in M.

Table A2

*Detailed Output for Monotonicity Check on Item T12P*

| **Group** | **Lo Score** | **Hi Score** | **N** | **F 0** | **F 1** | **Mean** | **P(X >= 1)** |  |  |  |
| --- | --- | --- | --- | --- | --- | --- | --- | --- | --- | --- |
| 1 | 2 | 7 | 98 | 36 | 62 | 0.6327 | 0.6327 |  |  |  |
| 2 | 8 | 8 | 94 | 51 | 43 | 0.4575 | 0.4575 |  |  |  |
| 3 | 9 | 9 | 123 | 63 | 60 | 0.4878 | 0.4878 |  |  |  |
| 4 | 10 | 11 | 110 | 73 | 37 | 0.3364 | 0.3364 |  |  |  |
|  |  |  |  |  |  |  |  |  |  |  |
|  | **#ac** | **#vi** | **#vi/#ac** | **maxvi** | **sum** | **sum/#ac** | **zmax** | **group** | **group** | **#zsig** |
| P(X >= 1) | 6 | 5 | 0.8333 | 0.2963 | 0.8889 | 0.1482 | 4.1296 | 1 | 4 | 4 |

Note that there are several decreases in the last column of Table A2. We can now compare these proportion-correct scores across the 4 different groups. So in this case there are 4*3/2 = 6 active comparisons (column ‘#ac’ in the output in Table A2).

Note that “a decrease between two subsequent lines increases in importance if it persists between non-adjacent lines” [4]. This is often the case in our example. Item T12P contains 5 violations larger than the default minimum violation (*minvi = .03)* among the 6 active comparisons, denoted ‘#vi’ in the output, with the largest violation (‘maxvi’) equal to 0.296 (the difference in proportion-correct between restscore groups 1 and 4). The 5 violations account for 83.3% of the total number of active comparisons (this is denoted ‘#vi/#ac’) in the output. So, a correct score on item T12P tends to imply a low score on the remaining 11 items. This is also reflected in the negative scalability coefficient for this item (ItemH = -0.14). Most of the remaining information in the last line of Table A2 can now be verified: The sum of the violations greater than *minvi* is 0.889 (column ‘sum’), which is, on average, 0.148 violations per active comparison (‘sum/#ac’).

To investigate whether the decrease in the proportion-correct between each two groups is due to sampling fluctuations, we can calculate the probability of exceedance using 2*2 tables of observed frequencies for each comparison. In our example, when comparing restscore groups 1 with 2 and 1 with 4, we obtain the following frequency tables:

Table A3

*Examples of 2*2 Tables Used for Assessing Significance of Violations of Monotonicity*

| Restscore groups 1 and 2 | | | | |  | Restscore groups 1 and 4 | | | | |
| --- | --- | --- | --- | --- | --- | --- | --- | --- | --- | --- |
|  | Group | F 0 | F 1 | Total |  |  | Group | F 0 | F 1 | Total |
|  | 1 | 36 | 62 | 98 |  |  | 1 | 36 | 62 | 196 |
|  | 2 | 51 | 43 | 94 |  |  | 4 | 73 | 37 | 188 |
| Total |  | 87 | 105 | 192 |  | Total |  | 174 | 210 | 384 |

Then, the probability of exceedance is the probability of 36 or less in the hypergeometric distribution with marginals 98, 94, 87, 105 which results, using a normal deviate to approximate this hypergeometric distribution, in a *z*-value of -2.2985. Using a default significance level of 0.05 one-sided, the critical value is *z^*^*=1.6449, this violation is statistically significant. Similarly, when comparing restscore groups 1 and 4, we find *z_14_* = -4.1396 which, in absolute value, is larger than *z^*^*, indicating that this violation is also statistically significant. The *z* values for the remaining three restscore group comparisons are: *z_13_* = -2.0213, *z_24_* = -1.6248, and *z_34_* = -2.2147. Significance is tested without a Bonferroni correction “because each significant violation in itself provides evidence against the model assumption of M” [4]. For the significance level 0.05, we therefore see that four out of the five violations are statistically significant (‘#zsig’ in Table A2). The largest *z*-value is 4.1396 (column ‘zmax’), found between restscore groups 1 and 4.

The *Crit* value summarizes the (weighted) information in the bottom line of Table A2 for each item and is given by [4]:

$$Crit_{i}=50*\left( 0.30-H_{i} \right)+\sqrt{\#vi}+100*{\#vi}/{\#ac}+100*maxvi+10*\sqrt{sum}+1000*{sum}/{\#ac}+5*zmax+10*\sqrt{\#zsig}+100*{\#zsig}/{\#ac}.$$

For item T12P, the *Crit* value equals 401.

1. **IIO Checks and the *Crit* Value. An Example**

Our example for IIO checks is based on a data set consisting of the responses of 828 individuals to a set of 17 items assessing coping strategies with industrial malodour [1] (also see [5]; data available within the “mokken” package [7, 8]). Each item has four ordered response categories, ranging from 0 (*never*) to 3 (*almost always*). For our purpose, we analyzed the dichotomized version of the polytomous data, with response categories 0 and 1 replaced by 0, and 2 and 3 replaced by 1. Similarly to the M assumption, we conducted the analyses with the default settings, that is, *minvi* was set to 0.03 and *minsize* was set to 82 [4]. Table B1 shows the summary table of the IIO checks for all items. The interpretation of Table B1 parallels that of Table A1. Again, note that when *#vi* = 0, *Crit* is automatically set to 0 both in the MSP5 and in the “mokken” software packages. Also, the *Crit* value resulting from application of the formula is rounded down to the nearest integer.

Table B1

*Summary Output for IIO Checks*

| **Item** | **ItemH** | **#ac** | **#vi** | **#vi/#ac** | **maxvi** | **sum** | **sum/#ac** | **zmax** | **#zsig** | **Crit** |
| --- | --- | --- | --- | --- | --- | --- | --- | --- | --- | --- |
| Item1 | 0.27 | 91 | 6 | 0.07 | 0.16 | 0.65 | 0.0071 | 3.22 | 4 | 81 |
| Item2 | 0.23 | 82 | 5 | 0.06 | 0.34 | 0.75 | 0.0092 | 5.44 | 2 | 107 |
| Item3 | 0.21 | 92 | 5 | 0.05 | 0.09 | 0.38 | 0.0041 | 1.56 | 0 | 39 |
| Item4 | 0.24 | 82 | 4 | 0.05 | 0.31 | 0.60 | 0.0073 | 5.14 | 2 | 98 |
| Item5 | 0.18 | 86 | 2 | 0.02 | 0.21 | 0.26 | 0.0030 | 3.79 | 1 | 68 |
| Item6 | 0.16 | 87 | 0 | 0.00 | 0.00 | 0.00 | 0.0000 | 0.00 | 0 | 0 |
| Item7 | 0.22 | 88 | 1 | 0.01 | 0.04 | 0.04 | 0.0005 | 1.54 | 0 | 20 |
| Item8 | 0.14 | 85 | 6 | 0.07 | 0.06 | 0.29 | 0.0034 | 1.38 | 0 | 39 |
| Item9 | 0.17 | 92 | 1 | 0.01 | 0.04 | 0.04 | 0.0005 | 1.54 | 0 | 22 |
| Item10 | -0.03 | 93 | 11 | 0.12 | 0.34 | 1.41 | 0.0151 | 5.44 | 5 | 147 |
| Item11 | 0.18 | 90 | 4 | 0.04 | 0.25 | 0.39 | 0.0043 | 4.48 | 1 | 81 |
| Item12 | -0.04 | 87 | 12 | 0.14 | 0.25 | 1.70 | 0.0196 | 4.48 | 8 | 151 |
| Item13 | 0.19 | 88 | 2 | 0.02 | 0.11 | 0.16 | 0.0018 | 1.93 | 1 | 46 |
| Item14 | 0.17 | 87 | 3 | 0.03 | 0.22 | 0.29 | 0.0033 | 3.78 | 1 | 72 |
| Item15 | 0.21 | 86 | 5 | 0.06 | 0.19 | 0.47 | 0.0055 | 3.73 | 2 | 78 |
| Item16 | 0.15 | 85 | 3 | 0.04 | 0.16 | 0.31 | 0.0037 | 2.33 | 2 | 66 |
| Item17 | 0.11 | 91 | 2 | 0.02 | 0.16 | 0.24 | 0.0026 | 3.22 | 1 | 63 |

The worst *Crit* value occurs for Item12, where 12 out of the 87 active comparisons, representing 14% of such comparisons, showed a violation greater than *minvi*. The largest violation for this item is *maxvi* = 0.25 and their sum is *sum* = 1.70. the largest *z*-value is *zmax* = 4.48, and 8 out of the 12 violations had a *z* value larger than the critical value *z^*^* = 1.645 corresponding to the significance level 0.05. The *Crit* value of 151 was estimated using the same formula as for violations of monotonicity.

An inspection of the item-pair plots shows that Item12 intersects with 9 other items. Table B2 shows the detailed output for the intersection of Item12 and Item11, which leads to the largest violation for Item12.

Table B2

*Detailed Output for IIO Check for Item Pair Item12 and Item11*

| **Group** | **Lo Score** | **Hi Score** | **N** | **Response combinations** | | | | **P(Item12 = 1)** | **P(Item11 = 1)** |
| --- | --- | --- | --- | --- | --- | --- | --- | --- | --- |
|  |  |  |  | **00** | **01** | **10** | **11** |  |  |
| 1 | 0 | 1 | 84 | 68 | 5 | 10 | 1 | 0.1310 | 0.0714 |
| 2 | 2 | 2 | 100 | 58 | 5 | 35 | 2 | 0.3700 | 0.0700 |
| 3 | 3 | 3 | 122 | 65 | 7 | 47 | 3 | 0.4098 | 0.0820 |
| 4 | 4 | 4 | 114 | 62 | 23 | 25 | 4 | 0.2544 | 0.2368 |
| 5 | 5 | 5 | 139 | 71 | 23 | 37 | 8 | 0.3237 | 0.2230 |
| 6 | 6 | 6 | 103 | 55 | 18 | 26 | 4 | 0.2913 | 0.2136 |
| 7 | 7 | 12 | 166 | 69 | 62 | 21 | 14 | 0.2108 | 0.4578 |
| Total |  |  | 828 |  |  |  |  | 0.2862 | 0.2162 |
|  |  |  |  |  |  |  |  |  |  |
|  | **#ac** | **#vi** | **#vi/#ac** | **maxvi** | **sum** | **sum/#ac** | **zmax** | **group** | **#zsig** |
|  | 6 | 1 | 0.1667 | 0.2470 | 0.2470 | 0.0412 | 4.4789 | 7 | 1 |

Table B2 shows that 7 groups have been formed based on the restscore on *I* – 2 = 15 items (excluding items 11 and 12). The restscore values 0 – 1 and 7 – 12 were joined in order to meet *minsize*. On the row marked ‘Total’, one can see that Item12 is overall more popular than Item11, as it has more positive answers (26.6%) compared to Item11 (21.6% positive answers). However, in the restscore group 7 the popularity is reversed: 0.21 is lower than 0.46 by 0.25 (calculated before rounding). Hence, out of 6 active comparisons, 1 (16.7%) showed a violation. Note that *#ac* = 6 not 7 in the bottom line of Table B2 because at least one of the restscore groups must have the same popularity order that was found overall.

In order to establish the statistical significance of the violation found in the restscore group 7, we need to inspect the 2*2 table of observed frequencies (column marked “Response combinations” in Table B2). If the popularity of Item11 is at most equal to the popularity of Item12, as indicated in the ‘Total’ row of Table B2, then the frequency for the response combination 01 (here 21 cases) is lower than the frequency in the response combination 10 (here 62 cases) purely by chance (McNemar’s test). The null hypothesis of equal popularities has an associated p-value (one-sided) equal to the probability of at most 21 successes in a binomial distribution of 21 + 62 = 83 trials with success probability 0.5. A *z­*-value of 4.48 can be obtained using a normal approximation for this distribution.

The same procedure is followed for the remaining item pairs that include Item12 and for the remaining items as well. Results are then added up for each item and summarized in a table similar to Table B1. The same rules of thumb proposed by Molenaar and Sijtsma [4] regarding the *Crit* index are used to evaluate the severity of IIO violations.

1. **Data-Generation Processes**

**Model-Fit Data**

Model-fitting item scores were generated according to the two-parameter logistic model (2PLM) [2]. We used this model to simulate item scores because it allowed us to obtain data that followed the specifications of each experimental condition. Moreover, the 2PLM is nested within the nonparametric models; thus, data that fit the 2PLM also fit the MHM. Furthermore, we could easily restrict 2PLM generated data to fit the DMM. The 2PLM is defined as follows:

|  | $\text{P(}\text{X}_{\text{i}}\text{=1\vertθ)=}\frac{\text{e}^{\text{α}_{\text{i}}\left( \text{θ-}\text{β}_{\text{i}} \right)}}{\text{1+}\text{e}^{\text{α}_{\text{i}}\left( \text{θ-}\text{β}_{\text{i}} \right)}}$ |  |
| --- | --- | --- |

In the equation above, *α_i_* denotes the discrimination of item *i*, reflecting how strongly the item is related to the person parameter *θ*, and *β_i_* denotes the difficulty or popularity of item *i*. To obtain data according to the DMM, we constrained the *a_i_* parameter to be equal across all items, leading to parallel IRFs. An overview of all the values we used throughout the study for the item and person parameters can be found in Table C1.

Table C1

*Item and Person Parameters for Each Simulation Condition*

|  | | Monotonicity violations | | | |  | Invariant item-ordering violations | |
| --- | --- | --- | --- | --- | --- | --- | --- | --- |
| Parameter | | None | Reversed IRFs | Quadratic IRFs | Unfolding IRFs |  | None | Intersecting IRFs |
| *α_i.Fit_* | |  |  |  |  |  |  |  |
|  | ^*)^*U*(1.5, 2.5) | ✓ |  |  |  |  | ✓ |  |
|  | ^**)^*U*(1.2, 1.5) | ✓ |  |  |  |  | ✓ |  |
|  | ^***)^*U*(0.7, 1.2) | ✓ |  |  |  |  | ✓ |  |
|  | *U*(1.0, 2.5) |  | ✓ | ✓ | ✓ |  |  |  |
|  | *U*(1.5, 2.0) |  |  |  |  |  |  | ✓ |
| *α_i.Misfit_* | |  |  |  |  |  |  |  |
|  | *U*(1.0, 2.5) |  | ✓ | ✓ | ✓ |  |  |  |
|  | *α_i.Fit_* ± 1 |  |  |  |  |  |  | ✓ |
| *β_i.Fit_* | |  |  |  |  |  |  |  |
|  | *N*(0, 1) | ✓ | ✓ | ✓ | ✓ |  | ✓ | ✓ |
| *β_i.Misfit_* | |  |  |  |  |  |  |  |
|  | *N*(0, 1) |  | ✓ | ✓ | ✓ |  |  |  |
|  | ^1)^*N*_trunc_(0, 1) |  |  |  |  |  |  | ✓ |
| *θ* | |  |  |  |  |  |  |  |
|  | *N*(0, 1) | ✓ | ✓ | ✓ | ✓ |  | ✓ | ✓ |
| *Note*: *α_i.Fit_* = discriminations for the model-fitting items; *α_i.Misfit_* = discriminations for the model-misffitting items; *β_i.Fit_* = difficulties for the model-fitting items; *β_i.Misfit_* = = difficulties for the model-misfitting items; *θ* = person characteristic; *U*(,) = the uniform distribution; *N*(,) = the normal distribution.  ^*)^ Yielded medium and strong scales in the *I_misfit_* = 0 conditions.  ^**)^ Yielded weak scales in the *I_misfit_* = 0 conditions.  ^***)^ Yielded scales with *H* < .3 in the *I_misfit_* = 0 conditions.  ^1)^ Values were drawn from the truncated standard normal distribution, bounded between *min*(*β_i.Fit_*) and *max*(*β_i.Fit_*). | | | | | | | | |

**Model-Misfit Data Generation**

**Violations of the M assumption.** The monotonicity of the misfitting items (*I_misfit_* items in total) was violated by generating the corresponding item scores from a model other than the 2PLM, resulting in IRFs that were either nonmonotone or monotonically decreasing. We generated three types of such IRFs: reversed, unfolding, and quadratic IRFs, described below. The IRFs for the remaining (*I – I_misfit_*) items were generated using the 2PLM.

***Reversed IRFs.*** We first generated response probabilities for all items according to the 2PLM, and then we simply subtracted the probabilities of the *I_misfit_* items from 1 to obtain reversed IRFs. These IRFs were decreasing across the entire range of *θ*. Although, in practice, this is an extreme type of misfit (e.g., as a result of improper scoring), we generated this type of misfit to investigate the power of the *Crit* measure to detect extreme misfit.

***Quadratic IRFs.*** Here we followed the same procedure as in Tendeiro and Meijer [6]: The IRFs of the *I_misfit_* items were allowed to decrease for some range of *θ* values. We modelled this by estimating a quadratic function at *θ* < 2.5, connecting the points (*θ_min_*, .60 ), ((*θ_min_* +2.5)/2, .30), and (2.5, $P_{2PLM}(\alpha_{i.Misfit},\beta_{i.Misfit})$), where *θ_min_* is the minimum generated *θ* value in the sample and $P_{2PLM}(\alpha_{i.Misfit},\beta_{i.Misfit})$ denotes the 2PLM IRF evaluated at 2.5 for item *i*. More specifically, the IRFs decreased between *θ_min_* and (*θ_min_*+2.5)/2 and increased across the remaining range of *θ* values. In this study, the point of inflection (*θ_min_*+2.5)/2 (minimum of the IRF) ranged roughly between -1.5 and 0.0 on the *θ* scale. In the simulated data (using a standard normal distribution for *θ*), roughly between 7% and 50% of the simulees had a decreasing IRF.

***Unfolding IRFs.*** Item probabilities for the *I_misfit_* items were computed using the method that was also used to generate quadratic IRFs, after which we inversed the parabola by subtracting all probabilities from 1. These types of IRFs mimicked those from an unfolding model [3]. If such an item occurred in a scale, for roughly 50% to 93% of the simulees the probability of correct response or agreement would decrease as their level on the measured characteristic increased.

**Violations of the IIO assumption.** Violations of nonintersecting IRFs were generated by choosing discrimination parameters (*a_i.Misfit_*) for the *I_misfit_* items different from those for the remaining (*I - I_misfit_*) fitting 2PLM items (*a_i.Fit_*). For the *I_misfit_* items, *a_i.Misfit_* was set to either *a_i.Fit_* + 1 or *a_i.Fit_* – 1.

**References**

1. Cavalini, P. M. (1992). *It’s an ill wind that brings no good. Studies on odour annoyance and the dispersion of odorant concentrations from industries*. Unpublished doctoral disseratation. University of Groningen, The Netherlands.
2. Embretson, S. E., & Reise, S. P. (2000). *Item response theory for psychologists*. Lawrence Eribaum Associates, Inc.
3. Hoijtink, H. (1990). A latent trait model for dichotomous choice data. *Psychometrika*, *55*, 641-656. doi:10.1007/BF02294613
4. Molenaar, I. W., & Sijtsma, K. (2000). *MSP5 for Windows: A program for Mokken scale analysis for polytomous items-version 5.0* [Software manual]. Groningen, The Netherlands: IEC ProGAMMA.
5. Sijtsma, K. and Molenaar, I. W. (2002). *Introduction to nonparametric item response theory*, Thousand Oaks, CA: Sage.
6. Tendeiro, J. N., & Meijer, R. R. (2015). How serious is IRT misfit for practical decision-making? (LSAC Research Report Series; Vol. RR 15-04). Newtown, PA, USA: law school admission council (USA).
7. van der Ark, L. A. (2007). Mokken scale analysis in R. *Journal of Statistical Software*, *20*(11), 1-19. doi:10.18637/jss.v020.i11
8. van der Ark, L. A. (2012). New developments in Mokken scale analysis in R. *Journal of Statistical Software*, *48*(5), 1–27. doi:10.18637/jss.v048.i05
9. Verweij, A. C., Sijtsma, K., & Koops, W. (1996). A Mokken scale for transitive reasoning suited for longitudinal research. *International Journal of Behavioral Development*, *23*, 241–264. doi:10.1177/016502549601900115
10. **False Positive Rates and Power for *#zsig***

Tables D1 and D2 show the false positive rates and the power of a more conventional method of checking for violations of M and of IIO, that is, whether or not there are one or more statistically significant violations.

Table D1

*False Positive Rates and Power for #zsig for Violations of M. Values Shown are Percentages of #zsig Values Greater than 0*

|  | | ^a^ False positive rates: | | |  | ^b^ True positive rates (power): | | |
| --- | --- | --- | --- | --- | --- | --- | --- | --- |
| Type of violation | | *I_misfit_* = 1 | *I_misfit_* = 3 | *I_misfit_* = 5 |  | *I_misfit_* = 1 | *I_misfit_* = 3 | *I_misfit_* = 5 |
| Quadratic IRFs | |  |  |  |  |  |  |  |
|  | *N* = 100 | <0.1 | <0.1 | <0.1 |  | <0.1 | <0.1 | <0.1 |
|  | *N* = 500 | <0.1 | <0.1 | 0.1 |  | 5.2 | 3.4 | 2.3 |
|  | *N* = 1,000 | 0.2 | 0.1 | 0.1 |  | 18.0 | 11.4 | 8.1 |
| Unfolding IRFs | |  |  |  |  |  |  |  |
|  | *N* = 100 | <0.1 | <0.1 | <0.1 |  | 2.1 | 1.1 | 0.3 |
|  | *N* = 500 | <0.1 | 0.2 | 2.2 |  | 94.8 | 87.8 | 45.8 |
|  | *N* = 1,000 | 0.1 | 0.4 | 4.9 |  | 99.3 | 98.9 | 78.4 |
| Reversed IRFs | |  |  |  |  |  |  |  |
|  | *N* = 100 | <0.1 | <0.1 | 0.8 |  | 6.9 | 4.2 | 0.8 |
|  | *N* = 500 | <0.1 | 1.0 | 68.5 |  | 99.8 | 99.8 | 67.2 |
|  | *N* = 1,000 | <0.1 | 4.1 | 83.3 |  | 100.0 | 100.0 | 83.0 |
| ^a^ Values computed over the (*I – I_misfit_*) items.  ^b^ Values computed over the *I_misfit_* items. | | | | | | | | |

Table D2

*False Positive Rates (Top Panel) and Power (Lower Panel) for #zsig for Violations of IIO. Values Shown are Percentages of #zsig Values Greater than 0*

|  |  | | *N* = 100 | *N* = 500 | *N* = 1,000 |
| --- | --- | --- | --- | --- | --- |
| ^a^False positive rates | | |  |  |  |
|  | Scale quality | |  |  |  |
|  |  | Unscalable items | <0.1 | <0.1 | <0.1 |
|  | | Weak scales | <0.1 | <0.1 | <0.1 |
|  | | Medium-strong scales | <0.1 | <0.1 | <0.1 |
| ^b^Power | | |  |  |  |
|  | Number of violating items | |  |  |  |
|  | | *I_misfit_* = 1 | <0.1 | 1.8 | 5.8 |
|  | | *I_misfit_* = 3 | 0.2 | 18.0 | 33.8 |
|  | | *I_misfit_* = 5 | 0.3 | 29.8 | 52.0 |
| ^a^ Values computed over the *I* items in the *I_misfit_* = 0 conditions (9 conditions).  ^b^ Values computed over the *I_misfit_* items in the *I_misfit_* = 1,3,5 conditions (18 conditions). | | | | | |

1. **Density Curves for the *Crit* Value**


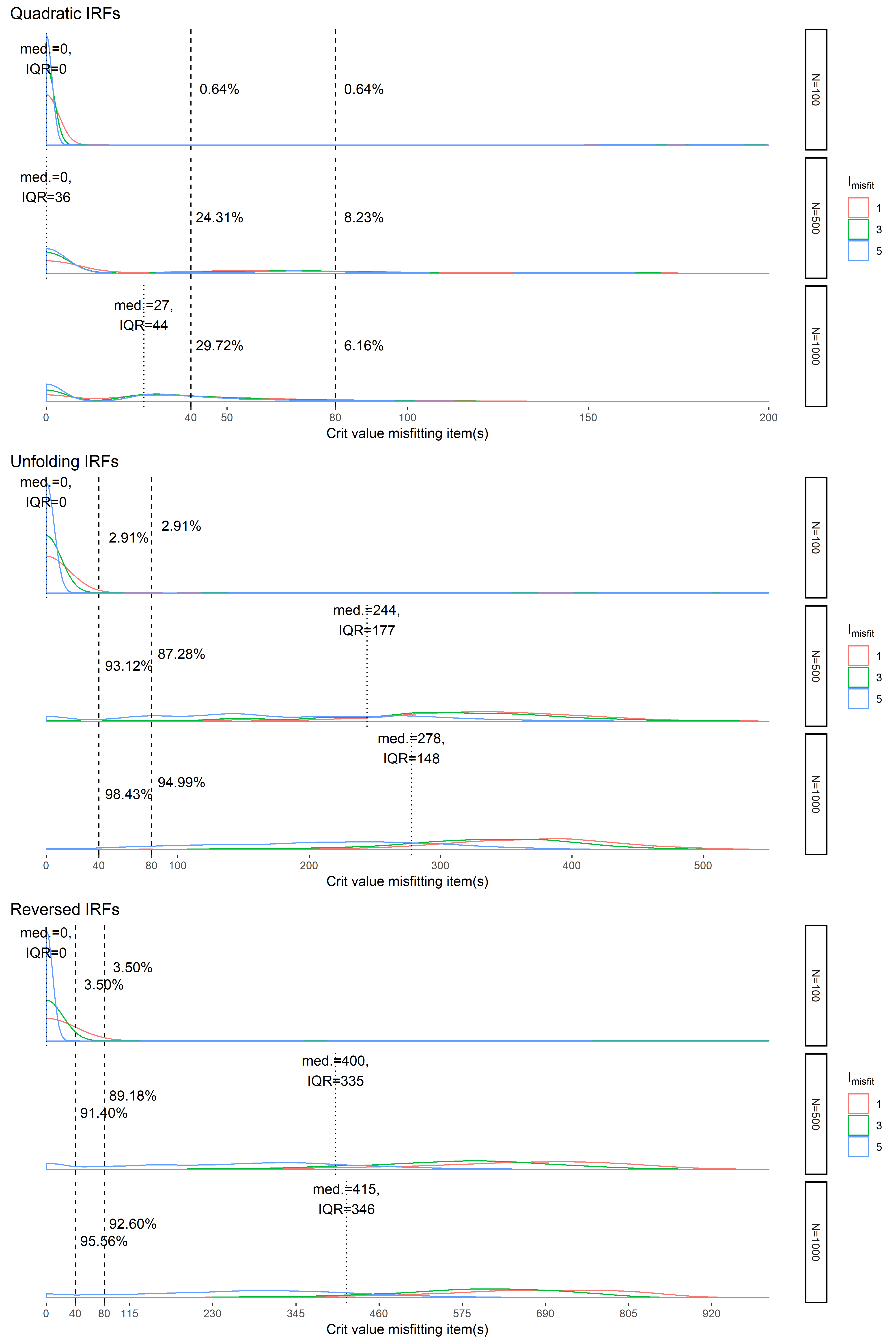


**Fig. E1** Density curves of the *Crit* value for evaluating monotonicity, corresponding to the misfitting items in the quadratic (top panel), unimodal (middle panel), and reversed (bottom panel) IRF(s) conditions. Percentages correspond to the *Crit* values at or above the often-used thresholds of 40 and 80. Dotted vertical lines and associated values denote the median Crit value and the interquartile range for each sample size. The power of *Crit* is superimposed on the density curves, separately for type of misfit and N. For extra detail, the plot also shows the percentage of *Crit* ≥ 40.


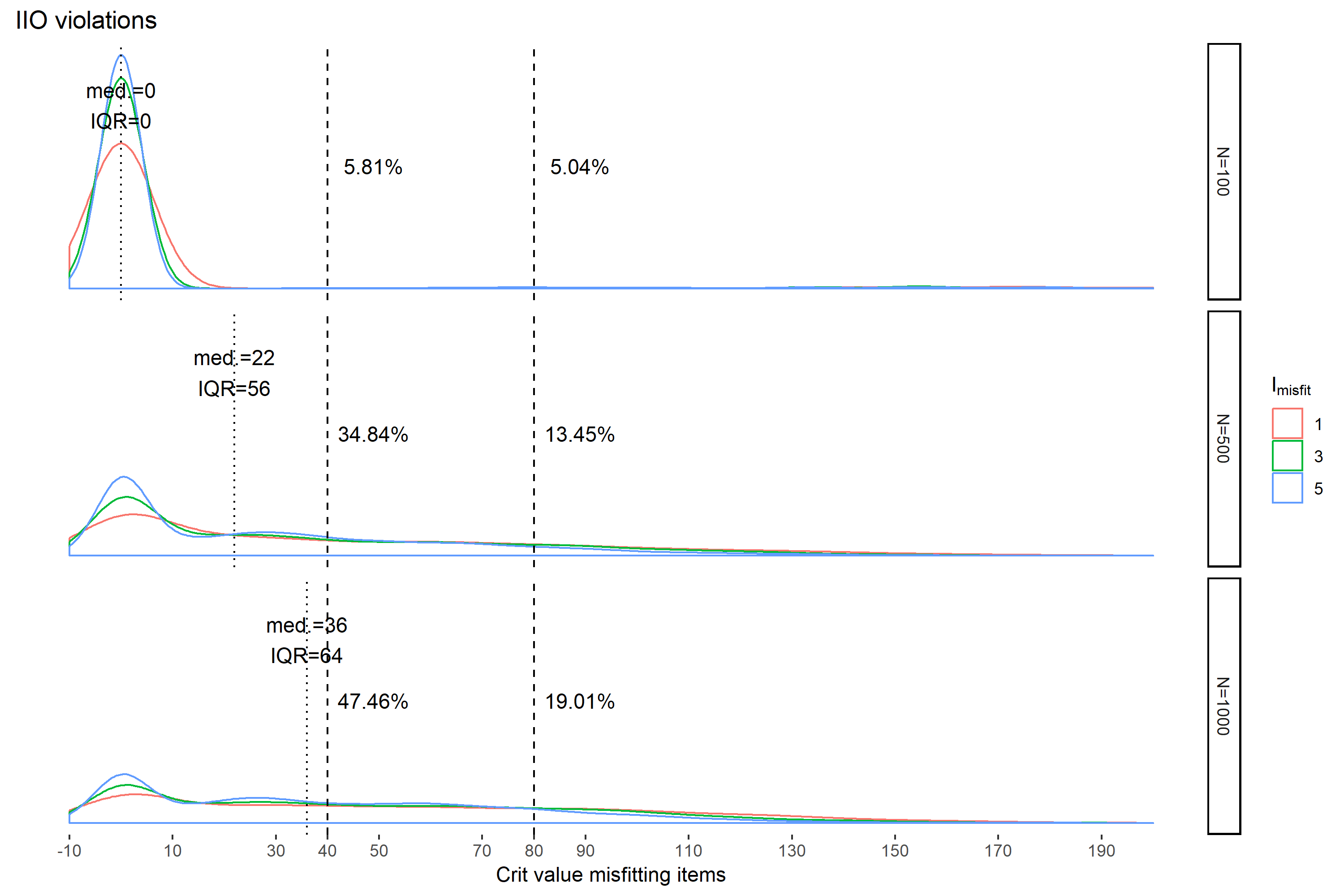


**Fig. E2** Density curves for the *Crit* values for checking the IIO assumption corresponding to the first Imisfit items. Percentages correspond to the *Crit* values at or above the often-used threshold of 80. Dotted vertical lines and associated values denote the median *Crit* value and the interquartile range for each sample size. The power of *Crit* is superimposed on the density curves, separately for type of misfit and *N*. For extra detail, the plot also shows the percentage of *Crit* ≥ 40.

1. We thank Don van Ravenzwaaij for sharing this insight with us. [↑](#footnote-ref-1)
